# Spatially resolved acyl transfer on surface by organo-catalytic scanning probe nanolithography (o-cSPL)†
†This article is dedicated to the memory of our friend and colleague Professor Teodor Silviu Balaban.
‡
‡Electronic supplementary information (ESI) available: Additional experimental data, blank experiments, DFT modeling, organic syntheses and procedures. See DOI: 10.1039/c8sc00294k


**DOI:** 10.1039/c8sc00294k

**Published:** 2018-04-16

**Authors:** Julien Botton, Katharina Gratzer, Cyril François, Vincent Mesquita, Lionel Patrone, Teodor S. Balaban, Sylvain Clair, Jean-Luc Parrain, Olivier Chuzel

**Affiliations:** a Aix Marseille Univ , CNRS , Centrale Marseille , iSm2 , Marseille , France . Email: olivier.chuzel@univ-amu.fr; b Aix Marseille Univ , CNRS , Univ Toulon , IM2NP , Marseille , France . Email: sylvain.clair@univ-amu.fr

## Abstract

Groundbreaking research done in the area of nanolithography makes it a versatile tool to produce nanopatterns for a broad range of chemical surface functionalization or physical modifications. We report for the first time an organocatalytic scanning probe nanolithography (o-cSPL) approach. Covalent binding of an organocatalyst on the apex of an atomic force microscope (AFM) tip gives way to a system that allows the formation of locally defined acylated-alcohol patterns on self-assembled monolayers (SAMs). With resolutions comparable to those of other cSPL methods, this first example of o-cSPL holds promise for future applications of bottom-up nanolithography set-ups employing this novel technique.

## Abstract 2

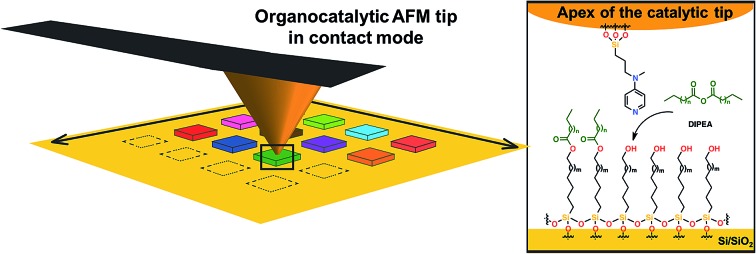
Local and catalytic acyl transfer for multipatterning of surfaces.

## Introduction

Over the last decades, the field of nanolithography^1^ has been developed to control materials at the atomic level.^2^ In addition to optical nanolithography,^3^ widely used in the semiconductor industry, multiphoton techniques,^4^ focused ion beam (FIB),^5^ or electron-beam (e-beam)^6^ have also appeared as promising tools to create two-dimensional (2D) nano-resolved patterns. More recently, nanoscale control has become possible by scanning probe microscopy (SPL),^7^–^12^ as for example with atomic force microscopy (AFM).^13^–^15^ This emergence has initiated the development of a wide variety of mask-free scanning-probe-based patterning methods, including dip-pen nanolithography, which has ascended as one of the most flexible ways.^16^,^17^ These techniques open new perspectives in bottom-up approaches, notably for organic substrates. With selective chemical transformations of self-assembled monolayers (SAMs) remaining challenging to date,^18^,^19^ catalytic scanning probe nanolithography (cSPL)^20^ has emerged as a promising and complementary tool among the various SPL techniques.^21^ The pioneering contributors to the field of cSPL have successfully focused their efforts on the use of catalytic probes coated with transition metals or their oxides.^22^–^31^ Nevertheless, robustness, reproducibly and leaching of catalyst material were weakly addressed in these studies. To extend the scope of cSPL using the large library of organometallic catalyzed reactions and to improve the above-mentioned points, our group has recently demonstrated that supported homogeneous catalysis could be a viable alternative.^32^ Indeed, the immobilization of a catalyst onto the apex of an atomic force microscope (AFM) tip allows performing selective covalent grafting of molecular objects on surfaces. This approach ensures a high nanoscale control by confining the chemical reaction at the interface of the catalytic tip and the surface. In this context, we recently reported the use of an immobilized 1,4,7-triazacyclononane (TACN) ligated manganese complex allowing the extension of the field of cSPL by performing a local epoxidation reaction. A second step of derivatization with piperazine or amine-based moieties was required to reveal the formation of nanostructures.^33^ Despite its intrinsic robustness, this catalytic system still suffers from some inherent drawbacks such as: (i) multi-step synthesis of the catalyst supported on the tip, (ii) the necessity of a post-functionalization step to “reveal” the topography of the new nanostructures, and finally, (iii) the impossibility to selectively graft more than one type of molecular objects during the “writing” process (catalysis and post-functionalization).

**Fig. 1:**
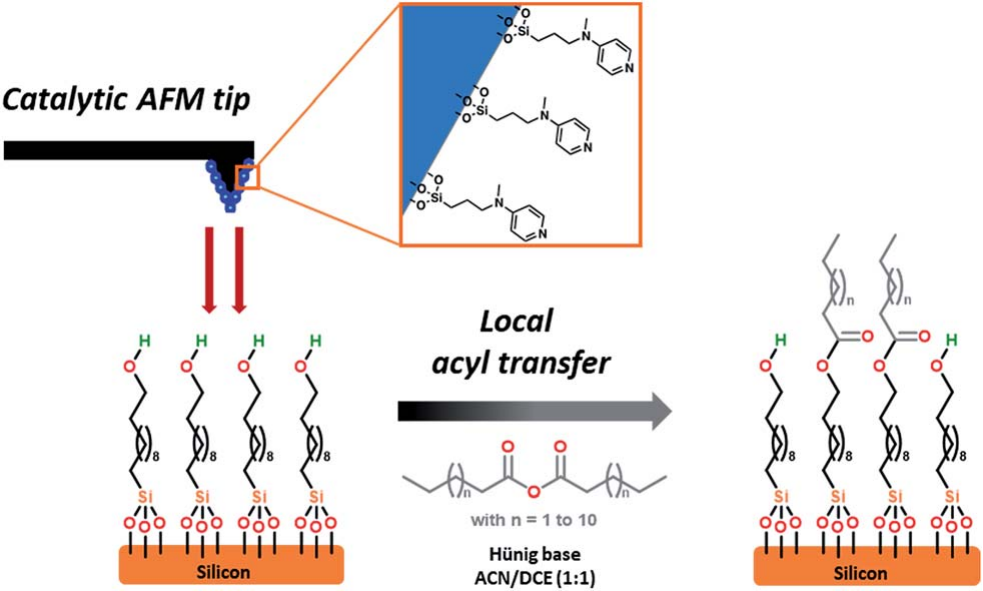
Schematic representation of the interface between the SAM-coated substrate and the AFM catalytic tip.

Aiming at simplifying the catalytic system even more, we hypothesized that an organocatalyst-supported AFM tip would constitute an attractive alternative. By avoiding the presence of any metal, potential “leaching” during the catalytic process is prohibited and more importantly, the stability of the catalytic system toward environmental parameters should be enhanced significantly (*e.g.* to air oxidation, moisture…). Herein, as depicted in Fig. 1, we report the development of a new catalytic system for SPL, based on a readily accessible aminopyridine catalyst scaffold. Due to their high importance in biological phenomena and their useful synthetic applications, acyl transfer reactions^34^ are among the most studied organic reactions in homogeneous phase. Based on the nucleophilic addition of the substrate (alcohols, amines, thiols) on an electrophilic acyl-donor partner (carboxylic acids, anhydrides, acyl halides), many catalytic systems for this reaction have been developed over the last century.^35^

Historically, *N*,*N*-dimethyl-4-aminopyridine (DMAP) derivatives, acting as nucleophilic organocatalysts,^36^ have been widely used as they enhance the electrophilic potential of the acyl partner, hence allowing rapid access to complex scaffolds.^37^ Those catalysts have shown their stability toward air and moisture and their silica immobilized versions are known to be readily available,^38^ making them promising candidates for cSPL.

We explored this approach by working on alcohol-terminated SAMs using myristic anhydride as the acyl source, demonstrating that SAMs can be locally acylated. The high degree of flexibility of the catalytic system has been demonstrated by grafting several distinctive patterns with a single tip as well as obtaining a high lateral resolution down to 45 nm, showing the potential of this AFM-driven organocatalysis.

## Results and discussion

**Scheme 1:**
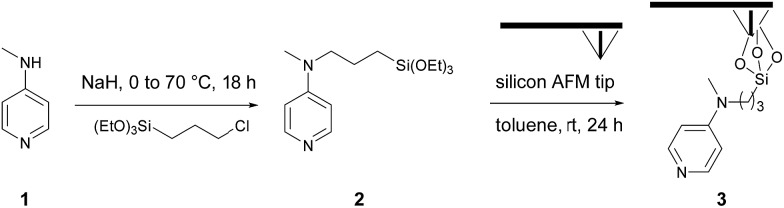
Two-step synthesis of catalytic AFM tip functionalized with an aminopyridine catalyst.

As part of a preliminary study, a homogeneous catalyst system was optimized at room temperature in chlorinated solvents (Table S1‡), requiring catalytic amounts of DMAP and stoichiometric amounts of myristic anhydride as acylating reagent and Hünig's base (*N*,*N*-diisopropylethylamine, DIPEA) as a non-nucleophilic base, and as a sacrificial proton sponge, to prevent any deactivation of the catalyst by trapping acidic by-products. The chosen catalytic system, is based on *N*-methyl-*N*-(3-(triethoxysilyl)-propyl)pyridin-4-amine **2**. This system was then evaluated in o-cSPL using an aminopyridine-functionalized AFM tip **3**. This catalytic probe was easily obtained in a two-step sequence by alkylation of *N*-methylpyridin-4-amine **1** with 3-chloropropyl-triethoxysilane, followed by its condensation on the AFM silicon tip (Scheme 1).‡ Whilst chlorinated solvents are the most commonly used for acyl transfer reactions in homogeneous catalysis, dichloromethane or chloroform were found to be too volatile to ensure full completion of the cSPL process (Fig. S1‡). After solvent optimization, a local AFM-catalyzed acylation was conducted by introducing a 50 mM solution of myristic anhydride and Hünig's base in a mixture of acetonitrile and dichloroethane (1 : 1) and by scanning a defined area in contact mode (writing mode). The modified area could be immediately observed in the topography image using AFM in tapping mode (reading mode).‡ To the best of our knowledge, this represents the first example of cSPL using organocatalysis as activation mode (o-cSPL).

**Fig. 2:**
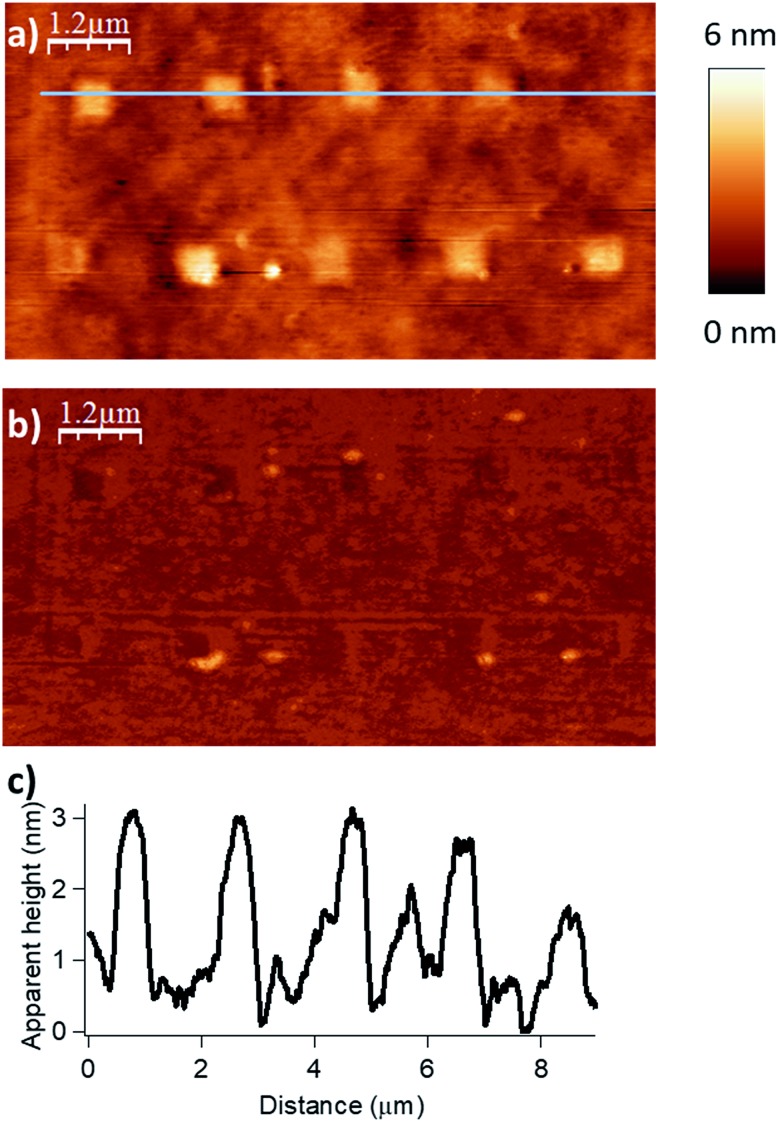
Influence of the applied force (*F*) on the esterification reaction with myristic anhydride as acylating reagent (*D*_IL_ = 8 nm, *V*_scan_ = 1 μm s^–1^). The applied force was incremented progressively from left to the right with respective values of 200, 1000, 2000, 2900 and 3800 nN for the upper line and 4800, 5700, 6700, 7600 and 9600 nN for the lower line. (a) Topography image in tapping mode after reaction. (b) Corresponding phase image. (c) Line profile (corresponding to the blue line in (a)) displaying an average height of 1.9–2.1 nm (theoretical calculated value of 1.78 nm in gas phase for a model, see Fig. S6 in the ESI‡).

**Fig. 3:**
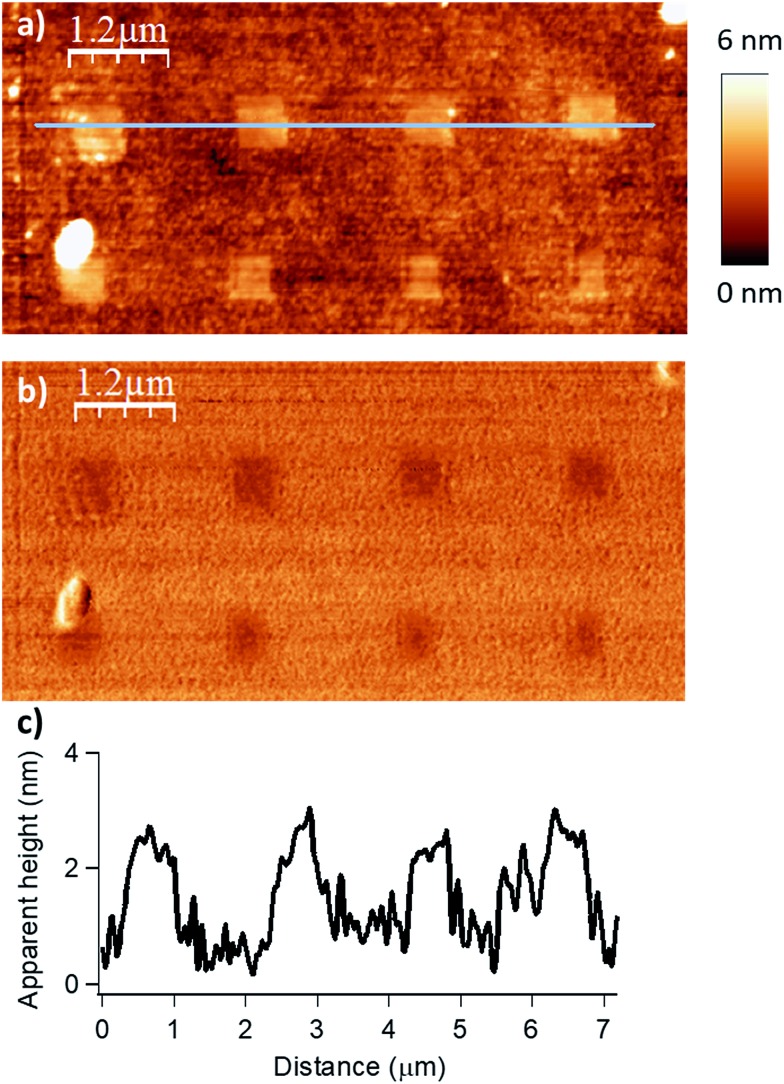
Influence of the scanning speed (*V*_scan_) on the esterification reaction with myristic anhydride as acylating reagent (*F* = 2900 nN, *D*_IL_ = 8 nm). The scanning speed was gradually increased from left to right with respective values of 0.25, 0.5, 1.0 and 1.5 μm s^–1^ for the upper line and 2.5, 5.0, 7.5 and 10.0 μm s^–1^ for the lower line of nanostructures. (a) Topography image in tapping mode after reaction. (b) Corresponding phase image. (c) Line profile (corresponding to the blue line in (a)) displaying an average height of 1.8 nm (theoretical calculated value of 1.78 nm in gas phase for a model, see Fig. S6 in the ESI‡).

We evaluated the robustness of the process and the influence of the different lithographic parameters on the local acyl transfer reaction from myristic anhydride as acylating reagent. The applied force (*F*), the scanning speed (*V*_scan_) and the interline distance (*D*_IL_) were varied over a certain range to produce 500 × 500 nm^2^ patterns as shown in Fig. 2 and 3.

The force parameter was evaluated first, and an array of nanostructures was obtained, with a *D*_IL_ of 8 nm and a scanning speed of 1 μm s^–1^ (Fig. 2).

The applied force was incremented progressively from left to right with respective values of 200, 1000, 2000, 2900 and 3800 nN for the upper line and 4800, 5700, 6700, 7600 and 9600 nN for the line below. For forces below 1000 nN, the lithographic process led to partial grafting. Forces above 1000 nN led to the formation of regular patterns allowing locally optimal defined acyl transfer reactions, especially, among the various attempts, with an apply force of 2900 nN. Moreover, the line profile (Fig. 2c) shows characteristic patterns with average heights of 1.9–2.1 nm, which match with the theoretical value of 1.78 nm calculated by DFT (Fig. S6 in the ESI‡). The high forces required are consistent with results obtained with related organometallic cSPL methodologies described previously.^33^

*D*_IL_ parameter was also evaluated. A *D*_IL_ of 4 nm did not show any structuration at all (Fig. S2‡), and a *D*_IL_ of 16 and 32 nm led to partial grafting (Fig. S3 and S4‡).

Then, the influence of the scanning speed on the catalytic process was investigated. The scanning speed was gradually increased from 0.25 to 10 μm s^–1^ applying a force of 2900 nN and a *D*_IL_ of 8 nm. This led to patterns of high density, resulting in an optimal range for grafting from 0.25 to 2.5 μm s^–1^ (Fig. 3). As previously, the line profile (Fig. 3c) shows characteristic patterns with an average height of 1.8 nm, which match very well with the theoretical value of 1.78 nm calculated by DFT (Fig. S6 in the ESI‡). It appears that the local acyl transfer is most efficient when the interaction between the catalytic tip and the surface is balanced by the proper lithographic parameters. The exceptional broad range of applied forces for which acyl groups are grafted on the surface, provides high robustness and stability to this “metal free” catalytic system and allows to tune it finely to prevent any undesired alteration of the surface.

**Fig. 4:**
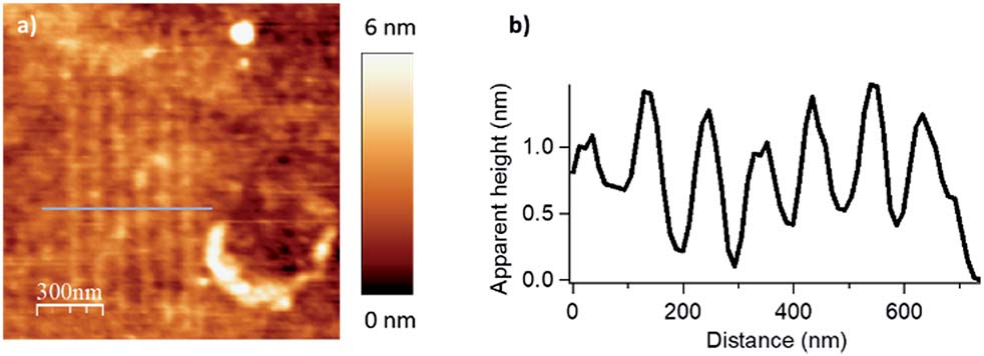
Resolution limit, for the esterification reaction with myristic anhydride as acylating reagent, with *F* = 2900 nN, *V*_scan_ = 2 μm s^–1^. (a) Topography image in tapping mode of an array of covalently attached nanostructures by catalytic local acyl transfer. (b) Line profile (corresponding to the blue line in (a)) displaying a lateral resolution down to 45 ± 5 nm (FWHM).

To evaluate the full potential in nanolithography of this catalytic system, individual parallel lines were scanned 100 nm apart under optimized writing conditions. The applied load was 2900 nN and the scanning speed was 2 μm s^–1^ with two passages by line procedure. The resulting patterns are presented in Fig. 4. A line width of 45 ± 5 nm (full width at half maximum) was achieved. Comparable to resolutions of 20–50 nm of other cSPL or dip-pen nanolithography techniques, this limit of lateral resolution has shown that this new catalytic system exhibits a high potential for the nanostructuration of surfaces.

**Fig. 5:**
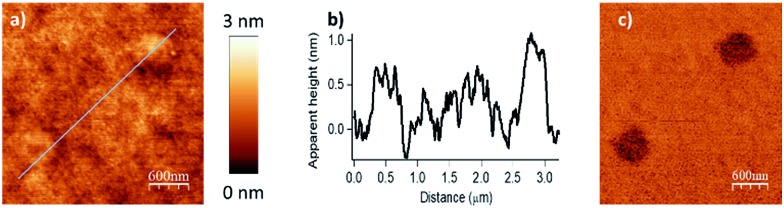
Surface patterning with multiple molecular objects (*F* = 2900 nN, *D*_IL_ = 8 nm, *V*_scan_ = 0.5 μm s^–1^). (a) Topography image in tapping mode of the esterification reaction with hexanoic anhydride as acylating reagent (bottom left corner), then with decanoic anhydride as acylating reagent (right top corner). (b) The line profile shows two distinctive patterns with an average height of 0.7 nm for the hexanoate ester pattern (theoretical calculated value of 0.75 nm in gas phase for a model, see Fig. S7 in the ESI‡) and 1.1 nm for the decanoate ester pattern (theoretical calculated value of 1.26 nm in gas phase for a model, see Fig. S8 in the ESI‡). (c) Corresponding phase image.

Finally, by consecutive grafting of two different molecular objects close to one another, with the same catalytic AFM tip, we demonstrate the large potential of our catalytic system. As shown in Fig. 5, patterns composed of two distinctive esters obtained from hexanoic anhydride and decanoic anhydride respectively, have been achieved by replacing the solution of one reactant by a fresh solution of another acyl donor to mimic a chemical flow reactor for local nanolithography. To ensure that no contamination occurred, several washing and control steps were realized between two injections. Then, only in the presence of the solvent mixture, a control pattern was realized using the conditions of the grafting procedure (*F* = 2900 nN; *D*_IL_ = 8 nm; *V*_scan_ = 0.5 μm s^–1^) to ensure the inertness of the catalytic probe. The reactor was then rinsed with a solution of decanoic anhydride, emptied and reinjected with a fresh 50 mM solution of decanoic anhydride and Hünig's base.§
§AFM experiments were carried out in a home-built Teflon liquid cell allowing facile and complete replacement of the solution inside without any sample shift using flexible plastic tubing and syringes (see ESI S8, Fig. S5‡). AFM measurements were performed in dichloroethane/acetonitrile (1 : 1) mixture (LC/MS quality, Fischer Scientific) inside a hermetic glass vessel under an air atmosphere saturated with dichloroethane/acetonitrile vapour in order to minimize solvent evaporation in the cell during long experiment times. Topographical imaging of the consecutive acyl transfer reactions, unambiguously shows the presence of two distinct well-resolved objects on the surface. The line profile shows two characteristic patterns with an average height of 0.7 nm for the hexanoate ester pattern (Fig. 5b, left), and 1.1 nm for the decanoate ester pattern (Fig. 5b, right), which are well fitting with the calculated values (0.75 nm and 1.26 nm in gas phase respectively, see ESI Fig. S7 and S8‡). This study clearly shows that this catalytic system is a powerful tool for the multiple functionalization patterning of surfaces.

## Conclusions

An innovative o-cSPL approach relying on the functionalization of an AFM tip with an organocatalyst has been achieved for the first time for the chemical nanostructuration of SAMs on Si/SiO_2_ under mild conditions. Our method of local acyl transfer allows to immediately observe topographical modifications of the substrate with covalent nanostructures without any need for post-functionalization. The catalytic process shows a high degree of robustness and flexibility leading to efficient multiple patterning of SAMs at the sub-100 nm scale. This time-efficient new nanolithographical process could exhibit great potential for high-throughput uses, as for example, toward mass-production through parallelization methods or alternative techniques.^39^–^43^ Interestingly, we demonstrated that the nanolithography can be selectively conducted with two distinct molecular reagents in a same experiment *via* a simple home-made flow-through reactor – this opening the door to future efficient nanoprinting with a large diversity of molecular “inks”. One-pot surface multi-functionalization could pave the way to the installation of different kind of molecular objects in close proximity potentially enabling communication between each other. All of that clearly establishes o-cSPL as a powerful tool for bottom-up nanolithography technologies.

## Conflicts of interest

There are no conflicts to declare.

## Supplementary Material

Supplementary information
