# Supplementary material for: Spatially resolved acyl transfer on surface by organo-catalytic scanning probe nanolithography (o-cSPL)
Source: Chem Sci. 2018 Apr 16;9(18):4280–4. doi: 10.1039/c8sc00294k (PMC5944244; doi:10.1039/c8sc00294k)
Supplement: Supplementary file 1 [file SC-009-C8SC00294K-s001.pdf]

Electronic Supplementary Material (ESI) for Chemical Science.
This journal is © The Royal Society of Chemistry 2018

Supplementary Information

Spatially Resolved Acyl Transfer on Surface by Organo-Catalytic Scanning Probe Nanolithography (o-cSPL)

Julien Botton,[a] Katharina Gratzer,[a] Cyril François,[a] Vincent Mesquita,[b] Lionel Patrone,[b] Teodor S. Balaban,[a] Sylvain Clair,*[b] Jean-Luc Parrain,*[a] and Olivier Chuzel,*[a]

[a.] Aix Marseille Univ, CNRS, Centrale Marseille, iSm2, Marseille, France
[b.] Aix Marseille Univ, CNRS, Univ Toulon, IM2NP, Marseille, France

* Corresponding authors: olivier.chuzel@univ-amu.fr, jl.parrain@univ-amu.fr and sylvain.clair@univ-amu.fr

Table of Contents:

1. Material and methods 2
2. Synthesis and grafting of the catalyst on silicon AFM tips 2
   2.1. Synthesis of a pyridine-type catalyst 2
   2.2. Grafting procedure on silicon AFM tips 3
3. Synthesis of the alcohol terminated SAMs on Si/SiO_2 3
   3.1. Preparation of a SAM of acetoxyundecylsiloxane 3
   3.2. Preparation of a SAM of 11-hydroxyundecyl)siloxane 4
4. Preliminary optimization of the catalytic system in homogeneous phase 4
5. Acyl transfer on surface by organo-catalytic scanning probe nanolithography 5
   5.1. Preliminary nanolithography results in chloroform 5
   5.2. Optimization of the lithography parameters 6
6. Flow reactor for AFM lithography 8
7. DFT modeling 8
   7.1. DFT modeling of the myristic ester on the SAM 8
   7.2. DFT modeling of the hexanoate ester on the SAM 8
   7.3. DFT modeling of the decanoate ester on the SAM 9

1. Material and methods

The AFM experiments were performed at room temperature (25 °C) with an Agilent 5500 microscope. AFM images were treated and analyzed using the WsXM software package.[1]

For all the experiments, AppNano ACT probes were used (cantilever length 125 µm, nominal frequency 300 kHz). The spring constant was calibrated by a thermal tune method on a Bruker Multimode 8 apparatus using a Nanoscope V controller and was evaluated to be 21 ± 1 N.m^{-1}. The pristine silicon tip has a nominal radius of curvature under 10 nm. The lithography process was obtained in proper reaction medium (liquid phase) using a tip that was previously grafted with the catalyst (see below). Delimited areas were scanned in contact mode with defined applied force and scanning speed. The characterization of the nanostructures was obtained in situ right after the reaction in tapping mode.

2. Synthesis and grafting of the catalyst on silicon AFM tips

2.1. Synthesis of a pyridine-type catalyst

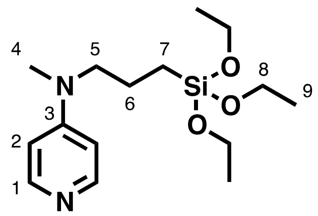

To a suspension of sodium hydride (355 mg, 14.8 mmol, 1.6 equiv) in THF (12 mL) was added dropwise at 0 °C a solution of *N*-methylpyridin-4-amine (1.00 g, 9.25 mmol, 1.0 equiv) in THF (28 mL). The reaction mixture was stirred for 2 h at room temperature, then a solution of 3-chloropropyltriethoxysilane (2.23 g, 2.2 mL, 9.25 mmol, 1.0 equiv) in THF (4 mL) was added dropwise at 0 °C. The resulting dark brown solution was heated at 70 °C for 20 h then cooled to room temperature. The crude mixture was filtered and concentrated under vacuum. {^{1}H}NMR analysis of the crude suggested the formation of the desired product. Purification by column chromatography ($CHCl_3$ on neutral alumina) afforded the desired product (596 mg, 1.90 mmol, 21%) as an orange oil.

^{1}**H NMR** (400 MHz, $CDCl_3$) δ 8.18 (d, ^3J = 5.1 Hz, $2H_1$), 6.48 (d, ^3J = 5.1 Hz, $2H_2$), 3.82 (q, ^3J = 7.0 Hz, $6H_8$), 3.45 – 3.23 (m, $2H_5$), 2.96 (s, $3H_4$), 1.69 (quint, ^3J = 13.4, 7.9 Hz, $2H_6$), 1.22 (t, ^3J = 7.0 Hz, $9H_9$), 0.67 – 0.51 (m, $2H_7$).
Data in accordance with the literature.[2]

2.2. Grafting procedure on silicon AFM tips

Catalytic AFM probes were obtained by coating commercial silicon tips with the appropriate pyridine-type ligand bearing an alkyl chain with a $Si(OEt)_3$ anchoring group. A flat bottom glass vessel (volume *ca.* 2.5 mL) containing a stirring bar was dried at 100 °C overnight, capped with a rubber septum and cooled to room temperature under argon atmosphere. Toluene (2 mL) and the pyridine-type catalyst (0.01 mmol) were added *via* a syringe and the solution was stirred for 10 min. The stirring bar was removed, and two AFM tips were placed on the bottom of the vessel and incubated under argon atmosphere for 24 h at room temperature. The resulting probes with grafted ligand were accurately washed with toluene then methanol (volume *ca.* 10 mL). Finally, AFM probes with the immobilized catalyst were dried under ambient conditions.

3. Synthesis of the alcohol terminated SAMs on Si/SiO_2

Based on the procedure developed by Tilman and co-workers,[3] the alcohol terminated SAMs on Si/SiO_2 were synthesized and characterized according to the following two-step sequence:

3.1. Preparation of a SAM of acetoxyundecylsiloxane

A piece of silicon wafer (1 x 0.5 cm^2) was put in a broad neck Erlenmeyer flask and sonicated in acetone for 10 min followed by deionized water for 10 min. Then, the wafer was immerged in a solution of Piranha (H_2O_2/H_2SO_4, 3:7) for 40 min at 115 °C, resulting in a gas release. The wafer was abundantly washed with deionized water starting from the tweezers, dried under a strong stream of argon and immediately immerged in a solution of acetoxyundecyltriethoxysilane (6 µL) in toluene (6 mL) for 2 h at 50 °C. The wafer was then rinsed with toluene before being sonicated for 15 min in toluene, 15 min in absolute ethanol and 15 min in deionized water. The wafer was dried under a stream of argon, slightly brushed with a paper tissue soaked with CH_2Cl_2 and stored at air. The

SAM formed was characterized by contact angle measurement with Milli-Qwater (48.0°).

3.2. Preparation of a SAM of 11-hydroxyundecyl)siloxane

Monolayers of acetoxyundecyltriethoxysiloxane on a silicon wafer were immersed at room temperature for 2 min in a solution of $LiAlH_4$ in THF (1.0 M from Aldrich), followed by 20% HCl aq. and large volumes of cold water followed by deionized water. The resulting monolayers were used without any further purification. The SAM formed was characterized by contact angle measurement with Milli-Q water (30°).

4. Preliminary optimization of the catalytic system in homogeneous phase with myristic anhydride as acylating reagent

The solubility of the anhydride partner (myristic anhydride), the yield of the desired acylated product and the absence of background reaction were evaluated on the following model substrate in homogeneous phase, to develop a suitable catalytic system for CSPL experiments.

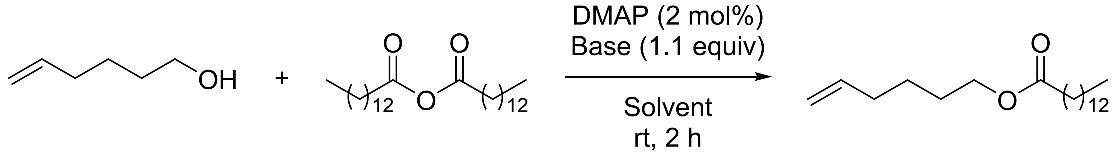

General procedure

To a solution of DMAP (2 mol%, 0.02 mmol), 5-hexen-1-ol (0.12 mL, 1 mmol) and Hünig's base (*N*,*N*-Diisopropylethylamine, DIPEA) (0.20 mL, 1.2 mmol) in solvent (2.5 mL) was added dropwise myristic anhydride (0.28 mL, 1.2 mmol) at room temperature and stirred for 2 hours. Then, methanol (0.5 mL) was added and the reaction was stirred for 15 min and the solvent evaporated. The resulting residue was dissolved in CH_2Cl_2 and washed with (NH_4Cl sat.). The crude mixture was extracted with CH_2Cl_2, dried over $MgSO_4$ and evaporated *in vacuo*, followed by purification *via* column chromatgraphy.

Table S1. Screening conditions for the design of the best catalytic system (using DMAP as a model catalyst and hex-5-en-1-ol as nucleophilic partner mimicking reactive SAMs).

Entry	Catalyst	Base	Solvent	Solubility of the anhydride	Yield (determined by ^{1}H NMR)
1	-	Et_3N	$CHCl_3$	High	30
2	-	DIPEA	$CHCl_3$	High	0
**3**	**DMAP**	**DIPEA**	**$CHCl_3$**	**High**	**quant.**
4	DMAP	DIPEA	DCE	High	0
5	DMAP	DIPEA	MeCN	Poor	quant.
6	-	DIPEA	MeCN	Poor	0
**7**	**DMAP**	**DIPEA**	**DCE/MeCN (1:1)**	**High**	**quant.**

5. Acyl transfer on surface by organo-catalytic scanning probe nanolithography

5.1. Preliminary nanolithography results in chloroform

The working area of the 11-hydroxyundecylsiloxane SAM on SiO_2/Si was first imaged in air in tapping mode with a catalytic tip. Then, local AFM-catalyzed acyl transfer was conducted by introducing a solution of 50 mM of myristic anhydride and 50 mM of the Hünig's base in chloroform, and by scanning a defined area in contact mode (writing mode). The modified area could be immediately observed in both, topography and phase images of AFM in tapping mode (reading mode).

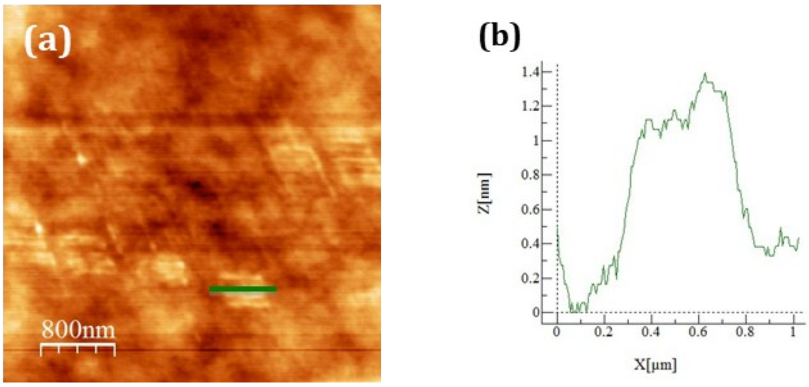

Figure S1. Preliminary results for local acyl transfer by o-CSPL. (a) Topography image after reaction in $CHCl_3$. The solvent evaporated along the experiment providing disrupted structures. (b) Line Profile of the pattern exhibiting the highest density.

Topography image (Figure S1a) after reaction in $CHCl_3$ showed that this solvent that led to full conversion under homogenous reaction conditions, is not directly compatible

with our catalytic nanolithography process. The solvent evaporated during the experiment leading to drifted structures that were difficult to spot on the topography image. Nonetheless, a line profile (Figure S1b) for one pattern displaying a sufficiently high density was obtained, making this a promising starting point for successful optimization studies.

5.2. Optimization of the lithography parameters

The working area of the 11-hydroxyundecylsiloxane SAM on SiO_2/Si was first imaged in air in tapping mode with a catalytic tip. Then, local AFM-catalyzed acyl transfer was conducted by introducing a solution of 50 mM of myristic anhydride and 50 mM of the Hünig's base in an acetonitrile (MeCN) and 1,2-dichloroethane (DCE) mixture (1:1), and by scanning a defined area in contact mode (writing mode). The modified area could be immediately observed in both, topography and phase images of AFM in tapping mode (reading mode).

Influence of force (F) and interline distance (D_{IL})

Figure S2 shows (D_{IL} = 4 nm) no correlation between topography and phase images. It is suspected partial nanoshaving or important reordering of the SAM structure as we previously observed in the AFM metal-catalyzed process.[4]

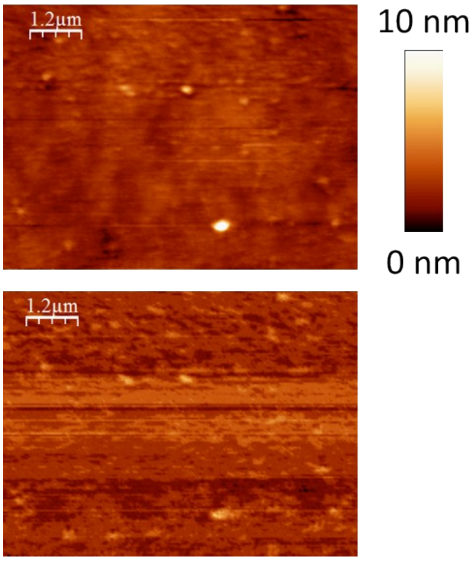

Figure S2. Influence of the force (F) and interline distance (**D_{IL} = 4 nm**) on the esterification reaction with myristic anhydride as acylating reagent (V_{scan} = 1 µm/s). Applied force was incremented progressively from left to the right with respective values of 200, 1000, 2000, 2900 and 3800 nN for the upper line and 4800, 5700, 6700, 7600 and 9600 nN. a) Topography image in tapping mode after reaction. b) Corresponding phase image.

In the case of D_{IL} = 16 and 32 nm, the lithographic process led to partial grafting (Figures S3 and S4).

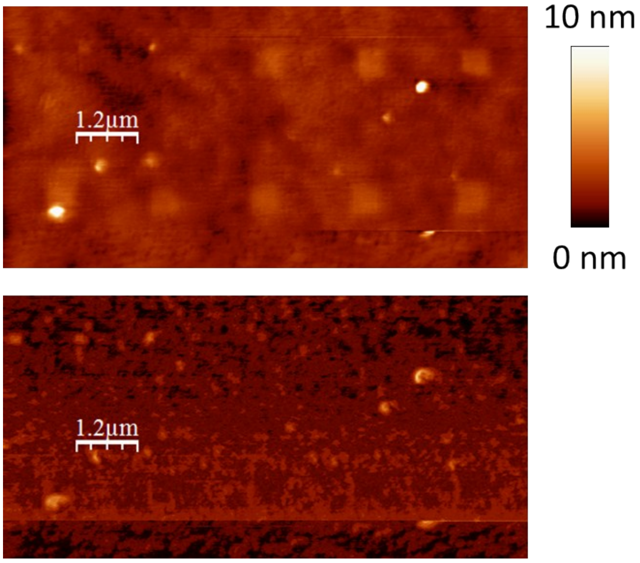

Figure S3. Influence of the force (F) and interline distance (**D_{IL} = 16 nm**) on the esterification reaction with myristic anhydride as acylating reagent (V_{scan} = 1 µm/s). Applied force was incremented progressively from left to the right with respective values of 200, 1000, 2000, 2900 and 3800 nN for the upper line and 4800, 5700, 6700, 7600 and 9600 nN. a) Topography image in tapping mode after reaction. b) Corresponding phase image.

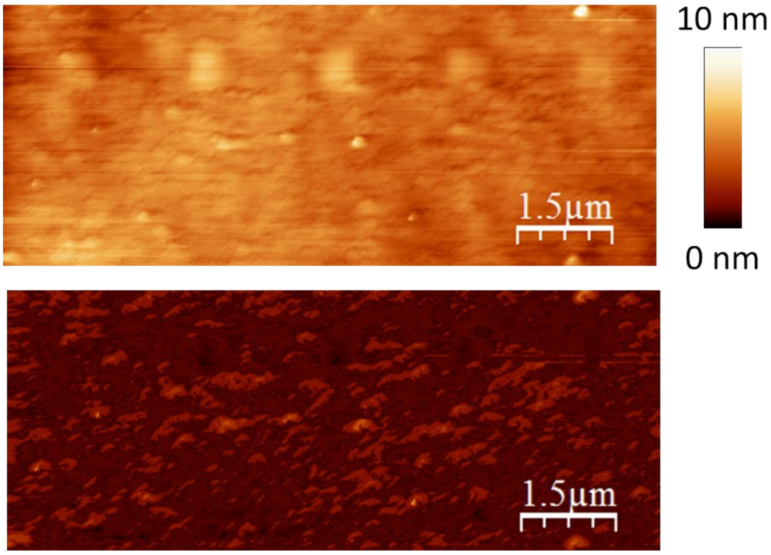

Figure S4. Influence of the force (F) and interline distance (**D_{IL} = 32 nm**) on the esterification reaction with myristic anhydride as acylating reagent (V_{scan} = 1 µm/s). Applied force was incremented progressively from left to the right with respective values of 200, 1000, 2000, 2900 and 3800 nN for the upper line and 4800, 5700, 6700, 7600 and 9600 nN. a) Topography image in tapping mode after reaction. b) Corresponding phase image.

6. Flow reactor for AFM lithography

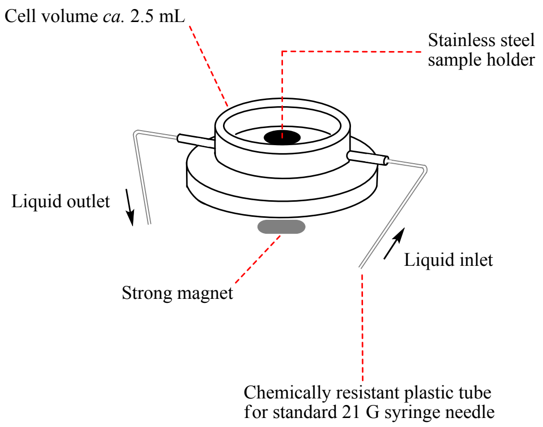

Figure S5. Schematic representation of the home-built AFM liquid cell.

7. DFT modeling

7.1. DFT modeling of the myristic ester on the SAM

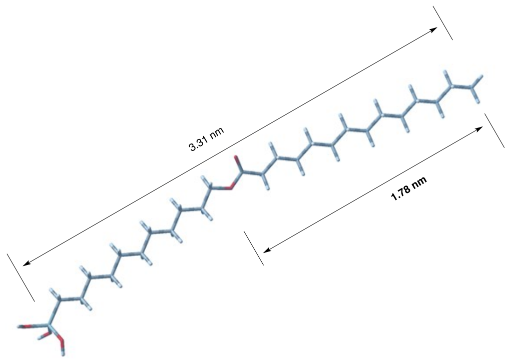

Figure S6. DFT calculations (B3LYP//6.31g(d,p), Gaussian 2009) of a model simulating the reaction between myristic anhydride and 11-hydroxy-undecanyltrihydroxysilane (model of the 11-hydroxy-undecanyl-siloxane SAM chain).

7.2. DFT modeling of the hexanoate ester on the SAM

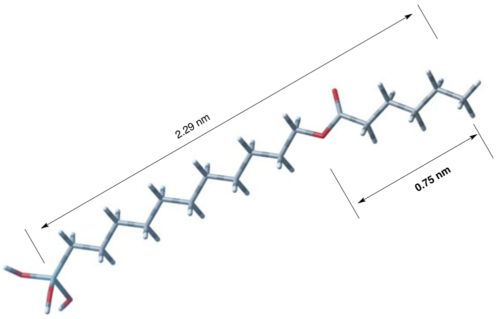

Figure S7. DFT calculations (B3LYP//6.31g(d,p), Gaussian 2009) of a model simulating the reaction between hexanoic anhydride and 11-hydroxy-undecanyltrihydroxysilane (model of the 11-hydroxy-undecanyl-siloxane SAM chain).

7.3. DFT modeling of the decanoate ester on the SAM

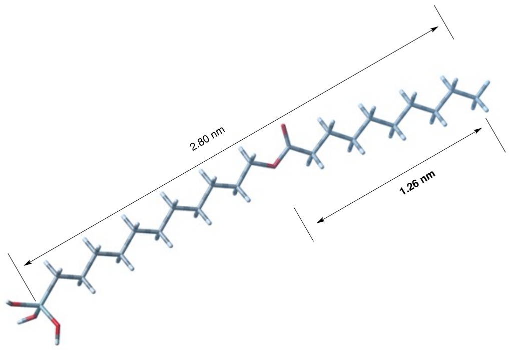

Figure S8. DFT calculations (B3LYP//6.31g(d,p), Gaussian 2009) of a model simulating the reaction between decanoic anhydride and 11-hydroxy-undecanyltrihydroxysilane (model of the 11-hydroxy-undecanyl-siloxane SAM chain).

1. Horcas, I.; Fernández, R.; Gómez-Rodríguez, J. M.; Colchero, J.; Gómez-Herrero, J.; Baro, A. M. *Rev. Sci. Inst.* **2007**, *78*, 013705.
2. a) Rubinsztajn, S.; Zeldin, M; Fife, W. K. *Macromolecules*, **1991**, *24*, 2682-2688. b) Chen, H-T.; Huh, S.; Wiench, J. W.; Pruski, M.; Lin, V. S-Y.; *J. Am. Chem. Soc.* **2005**, *127*, 13305-13311.
3. Tillman, N.; Ulman, A.; Penner, T. L. *Langmuir* **1989**, *5*, 101-111.
4. Mesquita, V.; Botton, J.; Valyaev, D. A.; François, C.; Patrone, L.; Balaban, T. S.; Abel, M.; Parrain, J.-L.; Chuzel, O.; Clair, S. *Langmuir* **2016**, *32*, 4034-404.
